# Motivations of assessment item writers in medical programs: a qualitative study

**DOI:** 10.1186/s12909-020-02229-8

**Published:** 2020-09-29

**Authors:** Sowmiya Karthikeyan, Elizabeth O’Connor, Wendy Hu

**Affiliations:** School of Medicine, Western Sydney University, Narellan Road & Gilchrist Drive, Campbelltown, NSW 2560 Australia

**Keywords:** Assessment, Item writing, Motivation, Faculty development, Quality assurance

## Abstract

**Background:**

The challenge of generating sufficient quality items for medical student examinations is a common experience for medical program coordinators. Faculty development strategies are commonly used, but there is little research on the factors influencing medical educators to engage in item writing. To assist with designing evidence-based strategies to improve engagement, we conducted an interview study informed by self-determination theory (SDT) to understand educators’ motivations to write items.

**Methods:**

We conducted 11 semi-structured interviews with educators in an established medical program. Interviews were transcribed verbatim and underwent open coding and thematic analysis.

**Results:**

Major themes included; responsibility for item writing and item writer motivations, barriers and enablers; perceptions of the level of content expertise required to write items; and differences in the writing process between clinicians and non-clinicians.

**Conclusions:**

Our findings suggest that flexible item writing training, strengthening of peer review processes and institutional improvements such as improved communication of expectations, allocation of time for item writing and pairing new writers with experienced writers for mentorship could enhance writer engagement.

## Background

Quality assessment ensures that students achieve intended learning outcomes. The negative impact of poor quality assessment items on learning is well known [1, 2], compromising the attainment of knowledge and skills required for safe and competent practice. Regularly producing sufficient quality items for use in written assessment is a continued problem for those responsible for delivering medical education. Developing new, robust assessment items requires content experts who have diverse teaching, research, clinical practice and administrative roles to also engage in item writing and has proved a perennial problem for medical program directors for which there is little explanatory research from which to design interventions [3].

Developing assessments is recognised as a key teaching competency for medical educators worldwide [4–6]. Standards set by the Association of American Medical Colleges recognises learner assessment as one of five key education activities to be undertaken by educators [5]. In the UK, assessment of learning is an essential domain in the Professional Standards for medical educators from the Academy of Medical Educators [7]. In Australia, the University Teaching, Criteria and Standards framework cites assessment and feedback as one of seven criteria to be achieved by all who teach in higher education [8], and medical school assessment collaborations have been created to share and benchmark quality examination and clinical assessment items [9–11]. The majority (78%) of deans, directors of medical education and academic chairs believe that assessment is a ‘very important’ competency for clinician-educators [12]. Yet Huwendiek et al. found that while medical educators perceived student assessment as an area of expertise, and poor assessment skills were a major challenge, it was only rated as the sixth most important area for further training [13]. The effectiveness of such institutional strategies in building assessment expertise for medical educators is weak [3] and illustrates a gap in the evidence.

Within medical schools, there is a range of teachers with formal and informal (e.g. honorary or unpaid clinician teachers) educational roles. While the latter could potentially contribute to item writing, their responsibilities may not be clear, and they may not identify as educators needing to meet professional standards. Furthermore, access to training may be limited due to their responsibilities being undefined. In some contexts, accreditation, professional standards or institutional requirements for professional development in assessment may not exist or be implemented [14]. Whether potential item writers should be expected to have such skills and produce items will vary across institutions, although the need to produce items is universal.

Bligh and Brice [15] identified that medical educators have varying responsibilities, and this variable accountability affects the generation of quality items. For instance, clinical supervisors may not be formally responsible for writing items yet feel invested in ensuring that students have acquired what is taught. Conversely, formal responsibility may not correlate with actual engagement in item writing.

Our previous research identified faculty development and quality assurance procedures as facilitators for good quality item writing [3]. There was a gap in the research directly exploring the factors that influence engagement with assessment writing and the impact of institutional policies and practices on teachers who write, and teachers who do not, but could write, items. Nor was this research informed by theory. We therefore sought to explore why item writers and potential item writers became engaged with item production with a theoretically informed study design, with the aim of informing transferable program improvements which could benefit many medical programs by reducing the effort required to generate items.

### Conceptual framework – self-determination theory

Our study was informed by self-determination theory (SDT), which identifies intrinsic and extrinsic motivation as fundamental principles that help explain human motivation [16].. Extrinsic motivation relates to external rewards while intrinsic motivation stems from an inherent interest or enjoyment. Being assigned formal responsibility could be regarded as extrinsic motivation to produce items, which may or may not be associated with intrinsic motivation, or an individual’s autonomous drive to produce items. According to self-determination theory, intrinsic motivation is driven largely by three factors – autonomy, competence and relatedness [16]. Autonomy refers to personal choice and self-determined decision making and behaviours. Competence is the self-perception of being able to successfully carry out a task or possess sufficient expertise in an area. Relatedness is the notion that shared goals or values create a sense of connectedness, or the pursuit of a higher goal [16].

These fundamental ideas of extrinsic and intrinsic motivation inform the model of controlled versus autonomous self-regulation [17, 18]. Extrinsic motivation can be further explained as a spectrum ranging from external regulation to integrated regulation. An individual’s compliance with a social rule or expectation can therefore be understood along a spectrum from compliance due to fear of repercussions or external rewards (external regulation), compliance due to fear of guilt or perceived pride from acceptance of the rule (introjected regulation), compliance due to comprehension of the underlying reasoning for the rule (identified regulation) to compliance due to the perception of the rule’s congruence with one’s own values (integrated regulation) [18–20]. Together, identified regulation, integrated regulation and intrinsic motivation form the concept of autonomous self- regulation, while external regulation and introjected regulation are merged to form the broad concept of controlled self-regulation [17, 18]. Applying this theory to the example of formal assignation of responsibility for item writing, individuals’ responses may range from producing items due to fear of repercussions such as negative performance review (external regulation) to producing items due to an inherent understanding of the value of effective assessment to learning and thus one’s responsibility as an educator (identified or integrated regulation). In medical education, SDT has previously been applied to research on factors influencing student engagement with learning, assessment and doing research, and teachers to teach [18, 21–23]. However, SDT is yet to be applied to item writer engagement.

Findings from qualitative research on the teaching motivations of clinicians and scientists who become medical educators [24–29] is relevant to the barriers faced by item writers, such as the challenge of balancing clinician or basic scientist roles with that of an educator [25, 26]. Browne et al. [25] recognised the acceptance of a change in role or status as a motivating factor to take up education; increased responsibility for assessment may prompt clinicians and academics to write assessment items but this task was not been specifically examined.

To address this unknown, we undertook a qualitative interview study with medical teachers to answer the question: what are the barriers and facilitators for current and potential item writers in medical schools to write good quality questions?

By exploring the motivations and experiences of individuals who currently write or could potentially write assessment items we sought to better understand the factors underlying the persistent challenge of producing sufficient quality items in medical schools in a theoretically informed way, with the aim of designing evidence-based strategies for engaging all who could write to produce quality assessment items.

## Methods

We report this study according to the COREQ reporting guidelines for qualitative research [30]. Adopting a constructivist stance, we conducted semi-structured interviews to explore the item writing experiences and intentions of academic staff and clinician teachers who currently or could potentially write items, and the factors that affected their engagement with the process.

### Study context

Interviews were conducted with academics and clinicians who taught in the 5 year undergraduate entry medical program at Western Sydney University, Australia. This relatively new medical school commenced in 2007 with a medical curriculum that was initially licenced from an established Australian medical school. The licence included a limited bank of items, including multiple choice, short answer and modified essay questions. However, a large proportion of items required de novo generation due to contextual and content differences in program delivery. The medical school participates in national assessment collaborations with other Australian and New Zealand medical schools to generate and share written items for benchmarking item quality and student performance. However, these collaborations result in only a very small proportion of questions used, and are used only for two examinations during the entire degree. The medical school does not licence questions from commercial item banks.

### Recruitment

Potential interviewees were all educators in the medical program. They were identified by their formal roles in the program to ensure that the entire range of roles and teaching settings, from course coordinators to small group tutors, from both on campus and clinical placement sites was sampled. We aimed for maximum variation in the degree of formal responsibility participants had for assessment. For example, appointed course coordinators or academic leads for defined components of medical program were deemed “responsible” for assessment. Small group tutors who facilitated and assessed substantial components of the course, who were not directly responsible for curriculum development or assessment were deemed “not responsible” but included as a separate participant group as they were potentially capable of writing items. Most of these tutors have medical or basic science backgrounds, with the rest having clinical and/or academic allied health professional backgrounds. These participant groups were included to address the study’s aim of investigating motivation to write items rather than previous item writing experience.

Participants were invited by email and subsequent participants identified by snowball sampling where interviewees were asked for potential participants who could have different views to their own. This was augmented by purposive sampling to ensure recruitment of participants with the defined characteristics and across clinical and basic science disciplinary backgrounds.

### Data collection and analysis

Interviews were conducted by SK between January 2016 and December 2018. Participants were aware that SK was a medical student at Western Sydney University who undertook this study as part of an Honours project.

Interview questions were informed by the factors identified by our previous study as those influencing engagement in item writing, with open ended questions (see Additional file 1). The questions were piloted with a non-participant teaching staff member. A semi-structured interview technique was used, with questions in subsequent interviews added to further explore themes identified earlier through preliminary analysis of participant responses.

Interviews were audio recorded and transcribed verbatim. Interview duration ranged between 19 min to 42 min. Participant data was anonymised prior to analysis. Field notes were made immediately after each interview to aid preliminary analysis.

Interviews underwent open coding and thematic analysis was performed according to Braun and Clarke [31]. Transcript excerpts were coded according to the overarching concept they highlighted. Coding of transcripts occurred concurrently with the collection with new interview data. When data collection was complete, all transcripts underwent repeat coding to ensure codes were congruent across the dataset. Codes were then transferred to an Excel spreadsheet for pattern analysis. Codes were grouped into categories which were used to inform the final themes. Initial open coding was undertaken by SK, and discussed with EO and WH to identify and develop themes for further sampling and analysis. Sampling continued until thematic saturation was reached, through iterative discussion of preliminary findings with all three researchers. Themes were further developed by constant comparison between participants with varying degrees of responsibility and engagement to identify similarities and differences. Final themes were agreed through iterative discussion with all researchers, generating descriptors which were revised and refined as the analysis proceeded.

Ethics approval was obtained from the Human Research Ethics Committee Western Sydney University, ID H9989. Written informed consent was obtained from all participants prior to commencement of the interview.

## Results

**Fig. 1:**
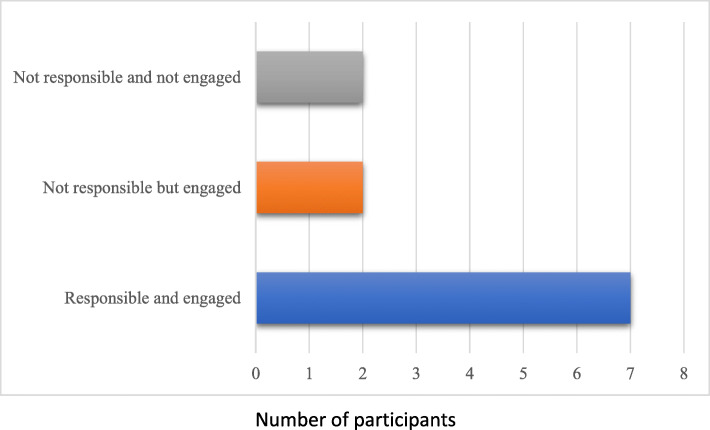
Participants’ responsibility and level of engagement in item writing. Responsibility was determined by the participant’s formally assigned roles. Engagement was determined by self-described involvement in item writing

Interviews were conducted with 11 educators in the medical program. Six were Problem Based Learning (PBL) tutors, 5 were university employed clinicians in academic teaching and research positions. Six were female. Five PBL tutors were employed in ongoing academic appointments, and one was employed on a sessional basis. Of the eleven participants, only two had received formal training in assessment. Participants described different levels of formal responsibilities and engagement in item writing (see Fig. 1). Formal responsibility was determined by their school assigned roles as coordinators of medical program components, or as lecturers and teachers who are formally invited to submit items. In contrast, PBL tutors may informally engage in item writing, but it is not a requirement of their teaching roles. Level of engagement was determined by the participant’s self-described involvement. Seven participants were classified as ‘responsible and engaged’, two were classified as ‘not responsible but engaged’ and two were ‘not responsible and not engaged’.

When asked the features of a good item, all described typical features such as being matched to important and clinically relevant knowledge, of appropriate difficulty and discrimination, covered what students were supposed to know, and clearly written.

Something that’s relevant. Something that they actually need to know. It’s worded well, so it’s not ambiguous. [Participant 6]

There were four major themes, comprising

- **Who’s Responsible?** or responsibility for item writing,
- Item writer **Motivations, Barriers and Enablers**,
- **How Much Expertise?** The content expertise required to write items and
- **Differences in the writing process**, particularly between clinicians and non-clinicians.

### Who’s responsible?

Participants who were ‘responsible and engaged’ referred to increased responsibility coming with a new academic role, prompting their increased engagement with item writing.

I think it’s one of the responsibilities I think you have as an academic, that that’s part of the package. [Participant 8]

When asked who they believed should be writing questions for medical school examinations, participants cited basic scientists who deliver or facilitate content in the medical program, and clinicians with their practice-based expertise. Some participants specified that only clinicians who held an academic position were responsible. Whether they were formally responsible or not, participants from the engaged groups highlighted the importance of ‘a team effort’ in generating sufficient items.

### Motivation, barriers and enablers

Participants reported a wide range of barriers and enablers which impacted on their motivation to write items. Key motivators included formal responsibility, the intellectual challenge of item writing, the importance of effectively assessing future doctors, and of testing content that writers were personally vested in.

It’s also quite a good intellectual challenge … it’s a constant upskilling of myself. [Participant 5]

It’s my job but it is very important … we are determining whether students pass and progress on or whether we fail them and whether they potentially move out of the medical program altogether … [Participant 1]

Less engaged writers who were less motivated to write cited a lack of time, multiple responsibilities and item writing not being a priority as key reasons that they were not more involved in the process.

I think that to be in involved in that process would take a lot of time which I don’t have right now, and I’d be interested in participating at some point, but probably not until I’ve finished my PhD. [Participant 2]

Other barriers and enablers are discussed as subthemes below.

### Social interactions and relationships

This subtheme relates to how interactions with peer writers and with students assisted with item writing.

Participants found peer review and feedback significantly facilitated item writing. While self-review was important, participants found that this skill needed time to develop and feedback from more experienced writers, particularly when they first began writing questions, was invaluable.

It’s important to have a peer review, so that if you’re writing a short answer question, that the question you’re asking, the intention of the question and the concepts, the way that it’s phrased, it’s clearly understood by others. [Participant 11]

Student interaction through teaching led to identification of content that students struggled with and thus ideas for items.

I think it’s just the interaction of seeing their thought processes, seeing whether they’ve actually grasped the knowledge, even though I’m not teaching them the knowledge … idea of you know, where their strengths are, where their weaknesses are … [Participant 4]

In addition to peer review, many thought the quality of the examination paper as a whole was improved by input from a variety of writers from different backgrounds. As well as the practicality of sharing the item writing workload for busy academics and clinicians with multiple responsibilities, a collaborative approach meant writers felt a sense of responsibility to contribute their fair share:

This is a team game and if I don’t do it … and then the load falls on one person or two people - (a) that’s not fair and (b) it doesn’t give a broad enough perspective on the subject [Participant 8]

### Organisational processes

Some did not recall being informed about the medical curriculum and existing database of items when initially recruited as item writers, nor when they were appointed to the university. For one writer based at the clinical school, this was a significant challenge:

I also don’t know - I don’t actually know what the curriculum is in general over the five years. So I don’t know what level people are meant to be at. [Participant 6]

There was no recall of being oriented as a new writer into the writing process, though some participants did receive resources and guidance from experienced writers when they asked for it.

Unpredictable scheduling of requests for new items was cited as a challenge. Those who were not formally responsible, and thus not overseeing the process, noted that items were often only requested near examination periods. Writing then needed to be urgently completed while still handling other responsibilities. While one participant felt that they were not given clear deadlines, another recalled receiving a deadline without much notice.

To sum, communication and administrative issues were barriers, particularly for those who felt engaged but were not formally responsible:

I didn’t readily have access to that, and I was unsure as to whether those lectures had been updated or not … I was unable to make contact with the lecturer. So, I think it’s a systems issue. Having the most recent lecture slides available, made available to the people that are going to create the exam questions, that’s very important. [Participant 11]

### How much expertise?

Competency in item writing requires both content expertise and writing expertise. While some participants suggested that item writing is a technical process and thus expertise could be facilitated by training measures, one participant stated that it is an intuitive process.

Participants across all three groups perceived content expertise as an important requirement and writing outside of one’s discipline was a major barrier for both engaged and unengaged writers. One ‘responsible and engaged’ writer suggested that clinicians could write outside their discipline due to having shared core medical knowledge and training at a level relevant for students. However, even writers with a broad range of content expertise still found the prospect of writing outside of their discipline challenging:

As a GP (general practitioner) it’s very easy for me to say oh look I’m a generalist I can write a variety of questions right across the board in lots of areas … but I think once you start writing exams it’s a completely different kettle of fish to, to doing that. [Participant 5]

One participant felt confident about writing outside of their discipline, provided there was access to information resources and the opportunity to work with content experts:

I think if I had the information, if I had information and if I was able to then check in with an expert in the field, then yeah, something I’d be capable of. [Participant 11]

More generally lack of confidence was a significant challenge for both current and potential item writers across all three groups. Despite much teaching experience, one potential writer cited a lack of specific item writing expertise as a key reason they had not been involved:

I think you need to put me in the category of even though I’ve had a lot of experience educating people, I haven’t had very much experience in generating the questions. [Participant 2]

Participants outside of the medical profession, such as those with an allied health background, also lacked confidence writing outside of their profession:

Because I don’t actually have a clinical background or a basic science background, so I probably wouldn’t be the best person to ask to write questions for the exams. [Participant 10]

### Differences in the writing process

In particular differences were described between medical clinician participants, and those from other disciplinary backgrounds. Clinicians found clinical experience a stronger inspiration for items than student interaction through teaching:

Probably less so, it’s probably more my clinical experience as being the part and parcel of writing questions rather than the actual PBL side of it. [Participant 5]

Medical clinicians were able to draw on knowledge about clinical medicine and experiences with patients to choose topics relevant to medical practice, but found it challenging to transfer this implicit knowledge into writing, using appropriate words:

You actually find yourself coming up with a question fairly easily on a certain subject for argument’s sake let’s say chlamydia but then trying to actually come with a very exacting question requires all the time. [Participant 5]

Alluding to unfamiliarity with the language of educators, these participants emphasised the importance of sharing a common language and understandings, by writing with those with whom they could easily communicate:

It’s really important that for clinicians you have someone who really speaks exactly the same language … to translate between sort of the medical education, the educational theory and the clinical language that you’re very comfortable with. [Participant 1]

Rather than collegiality and professional knowledge, those who were not clinicians tended to rely on their understanding of the taught curriculum and of standard item writing processes such as blueprinting in their approach to item writing:

“So, reviewing through lecture slides, tutorial information, and thinking about ensuring that I – ensuring for the students that the information that’s being delivered is what is being assessed is really important, and to try and have a spread across the curriculum and across the two topics, so that one particular component isn’t more heavily assessed than another.” [Participant 11]

## Discussion

Our theoretically informed approach to the enduring challenge of producing items has illustrated how item writers in a medical program perceive item writing, its processes, barriers and enablers, what they view as sufficient expertise required to be an item writer and the differences in approach between clinicians and others. Our findings appear to map well to self-determination theory concepts, identifying extrinsic and intrinsic motivators and suggesting practical measures support and engage item writers (see Table 1). Similar to students, teachers’ motivation will influence outcomes in medical education [18, 32], and supporting autonomy and relatedness may increase intrinsic motivation [23].

**Table 1:** Application of themes and subthemes to Self-Determination Theory and the practical implications

Motivation	Illustrative Theme	Illustrative subtheme/quote	Practical implication
Extrinsic motivation	Who’s responsible?	Because I was asked	Item writing clearly specified as role for teaching academics and clinicians. Training informed by shared values of delivering quality education, and thus the need to necessity produce quality assessment items
Intrinsic motivation			
• Autonomy	Barriers (System) Enabler	It’s not a priority It’s time consuming Because I was asked Organisational processes I enjoy writing about this topic/being able to select topics that I enjoy writing on	Improved communication of expectations, allocation of time for item writing, recognition of contributions. Scheduling of requests for items, acceptance of items throughout the year.
• Competence	How much expertise Enabler Clinician/non-clinician Barriers	Writing outside of discipline Certainty about what to assess Content expertise Organisational processes Feedback (lack of)	Increase item writing expertise – training in various and flexible formats to suit busy clinicians Processes to induct new writers to university curriculum, given examples of previous items, directed to item writing guidelines Procedures for feedback to writers
• Relatedness	Enablers Barriers	Link with future practice Student interaction Peer review It’s a team effort Mentorship Non-medical health background Based at clinical school	Informal and formal processes for peer review New writers are paired with experienced writers for peer support and review Item review meetings and peer group discussionss Support medical educators from different backgrounds to write items through item writer training, access to content experts for consultation at clinical school

Previous studies have explored the motivations of people in becoming medical educators [27, 28]. Our findings suggest that exploring the motivations of medical educators to write items could inform measures to foster autonomous motivation and engagement in the assessment process. While most participants alluded to extrinsic motivation – the formal responsibility to produce assessment items or being asked to by colleagues who were responsible – in prompting their engagement with the writing process, we found that this often coexisted with intrinsic motivators such as the opportunity to upskill and the intellectual challenge posed by the task. The ability to write items about topics participants enjoyed appeared to further promote autonomy. Shifting educators’ extrinsic motivations from external regulators to these more internal regulators could further promote autonomous self-regulation [17]. As an example, some participants who cited formal responsibility to write items as a motivator also recognised the importance of assessment (identified regulation), and one (Participant 1, p12) implied integrated regulation rather than external regulation.

Increasing competence, the self-perceived ability to successfully write a good quality item, is also an important consideration when developing measures to support item writers. While clinicians were equipped with implicit knowledge through their medical practice, they found it challenging to transfer this content into an assessment item, perhaps due to a lack of item writing experience or training. For clinicians, personal clinical interests shaped the items they wrote, partly due to confidence in their area of expertise, that is, self-perceived competency. Certainty about what to assess contributed to competency, particularly for non-clinicians. Poor communication and lack of organisational processes that supported competency for all writers posed a challenge.

The pursuit of a shared goal, or relatedness, to appropriately assess medical students to ensure that they become competent doctors also served as intrinsic motivation for item writers. For clinicians, writing items provided a link to future practice and developed their investment in the training of future clinicians. Student interaction and formation of professional relationships through peer review further enhanced relatedness. Lyness et al. described the importance of facilitating relatedness for faculty teaching through creating appropriate institutional structures [21]. Improved structures for collaboration between medical educators from different fields could potentially improve the number and scope of items generated. Lyness et al. further suggests practical implications for increasing motivation of medical educators to teach [21]. These strategies have been used to inform suggestions to foster intrinsic motivation to write assessment items (see Table 1).

### Practice implications

Our findings have highlighted the challenges of engaging academics and clinicians who are time poor and not based at the university with training workshops and suggests a range of theoretically informed practical strategies (see Table 1). However, there is significant evidence that faculty development programs do improve item quality in medical school examinations [2, 33–37]. Moreover, studies adopting an SDT analysis such as Lyness et al. have previously highlighted the importance of supporting the development of skills necessary to meet the tasks given to medical academics [21]. Providing training in various and flexible formats to suit busy clinicians, such as online or video sessions that could be viewed at their convenience, is therefore a key measure to train new writers, as well as existing writers who may not have previously received formal training. Allowing writers the autonomy to utilise training sessions at their own leisure thereby giving choices [21, 23] could increase engagement and thus improve the effectiveness of these training measures.

The importance of peer review processes in improving item quality has been established by numerous studies [38–40], and was found by our study to facilitate item writing through creating a sense of relatedness amongst writers. This is supported by findings from Lyness et al. who suggested that an important measure in supporting relatedness amongst academics is to create structures that cultivate interpersonal relationships within this group [21]. Thus, strengthening peer review processes in medical schools is an important practical implication.

Organisational improvements could include more streamlined communication of deadlines and expectations for writers, with a focus on regular spacing of requests for items. A clear process for orientation to the university curriculum and item writing methods for newly recruited writers would further increase self-perceived competence. Medical schools could support relatedness through fostering individual relationships [21] by pairing new writers with experienced writers who could act as mentors and provide guidance when required.

Uncertainty regarding responsibility for item writing could be resolved through clearly specifying item writing as a role for teaching academics and clinicians in a medical program. In this study context it appears that targeting both extrinsic (e.g. clearly communicated roles that are reinforced) and intrinsic motivations (e.g. desire to produce good doctors) together would be most effective in engaging writers. Providing evidence of the relationship between quality assessment and ensuring achievement of graduate learning outcomes to item writers could be one way for faculty trainers to further promote autonomous self-regulation.

Other strategies proposed by participants included the use of post-assessment student feedback and recruiting alumni and other junior doctors as item writers or reviewers. Such measures have already been employed by some medical schools as part of efforts improve item quality, including Maastricht University’s use of student feedback as part of their progress testing [41]. Cross disciplinary efforts such as between academics and clinicians or basic scientists and clinicians, as well as collaboration with other medical schools were also suggested by participants. Supporting medical educators from allied health backgrounds to write items through item writer training and access to content experts for consultation is a further practical implication. Additionally, developing a system to provide feedback on item performance to individual writers could further improve item quality.

### Limitations

A key limitation was that we were unable to recruit participants who described themselves, or were described by others, as ‘Responsible but not engaged’ in item writing. This is not surprising since assignation was largely on the basis of self-description and socially desirable answers are likely. Furthermore, the interviewer being a medical student may have led participants to discuss their experiences in a more positive light. We were only able to informally corroborate participant self-assignation with course coordinators’ nomination of potential participants through purposive sampling. The disengaged group perhaps creates much of the continuing challenge of writing enough quality items. This phenomenon is likely to affect other medical schools, resulting in questions being written by a select group or even one individual, with the motivations of the unengaged being largely un-researched.

Our findings are limited to those who are engaged, and those who are yet to write, but are not formally responsible. However, they illustrate how medical schools could better support writing and the motivation to write in these potential contributors. Focussing efforts on supporting this group could be more effective than expending efforts on those who are not motivated at all to write.

While some cited various system barriers to full engagement, participants who were formally responsible did contribute substantively to item writing. Whether formally responsible or not, our results, and their fit with SDT, suggest ways in which intrinsic motivation can be promoted in those who are disengaged with item writing.

### Gaps for future research

Future research could focus on sampling the formally responsible but disengaged or uninvolved writer and whether the phenomena described in our study are confirmed in other medical schools. Rather than targeting one group, testing the effect of the interventions suggested by SDT to better engage potential and actual item writers, as well as addressing the educational management and leadership aspects of assessment delivery may lead to more overall benefits.

## Conclusions

Our study has demonstrated key barriers and enablers to writing good quality items for individual writers, as well as perceptions of the level of expertise required for potential item writers. SDT has previously been applied in medical education to promote learning and research in students [22, 23, 32, 42], and teaching in medical educators. This study has adopted an SDT framework to analyse the challenges faced by medical educators in writing quality items and to inform evidence-based suggestions for improving item quality. This may be a further area for researching the effect of SDT informed interventions, not only in assessment and item writing. Designing and testing faculty training in item writing and assessment that is informed by SDT using design-based research principles may better encourage the production of sufficient quality items for medical programs.

## Supplementary information

**Additional file 1.**

## Data Availability

Data will be stored in an institutional data repository Research Direct https://researchdirect.westernsydney.edu.au as per institutional policy.

## Abbreviation

SDT: Self-Determination Theory
