# Supplementary material for: Motivations of assessment item writers in medical programs: a qualitative study
Source: BMC Med Educ. 2020 Sep 29;20:334. doi: 10.1186/s12909-020-02229-8 (PMC7523313; doi:10.1186/s12909-020-02229-8)
Supplement: Supplementary file 1 — Additional file 1. [file 12909_2020_2229_MOESM1_ESM.docx]

**Motivations of assessment item writers in medical programs: A qualitative study**

**Appendix 1: Interview questions**

1. To start with, could you please describe your experiences, so far with writing and developing exam questions? ***If yes:***
   1. How long have you been writing questions for?
   2. Have you written questions or been involved with the question writing process continuously during this period?
      - If they stopped: Why did you stop writing questions in that period?
      - If they continued: What motivated you to continue writing questions?
   3. For which students/trainees?
   4. On what topics?
   5. What roles and tasks have you undertaken in question development? (writing questions, reviewing and revising questions, marking, standard setting, psychometric analysis…)

*For current writers:*

1. Tell me about how you go about writing questions. Can you please describe the last time you wrote a question?
   1. What was the question about? Who was it for?
   2. How did you come up with the idea?
   3. What did you actually do when writing the question?
   4. How long did it take you? How did you find the time to write it?
   5. What were the challenges to writing this question?
   6. What would have made it easier for you?
2. What prompted you to begin writing questions? What interests you in writing assessment questions? What motivates you to keep writing questions?

*For ALL participants:*

1. What are the challenges for you in writing exam questions?
2. What would make it easier for you to start writing/write more exam questions?
3. To you, what is a good quality exam question?
   1. Can you think of any example, and describe its features?
4. Have you undertaken any training or received support or information about writing exam questions? ***If yes:***
   1. What was it?
   2. What was the duration and how long ago?
   3. What did you find useful about the training, if anything? How have you used this?
5. In your opinion, who should be writing questions for medical school examinations? (Academics, Clinicians, Lecturers, Associates of the SOM, PBL Tutors, etc.)
6. Who do you think we should interview that may have a different opinion to you on this subject? AND/OR

Who do you think we should interview that is a potential but not current question writer?

To help us analyse the data, I would like to ask about you. Please note that this information will not be used to identify you.

- 1. What is your position title? (Professor, Associate Professor, Senior Lecturer etc)
  2. What is your discipline?
  3. When did you start working at this medical school?
  4. When did you start teaching medical students?

1. Do you have any last points about writing questions, what makes it challenging, what (would) make it easier for you?
